# Co-transfection of hepatocyte growth factor and truncated TGF-β type II receptor inhibit scar formation

**DOI:** 10.1590/1414-431X20199144

**Published:** 2020-01-13

**Authors:** Ji-Hua Xu, Wan-Yi Zhao, Qing-Qing Fang, Xiao-Feng Wang, Ding-Ding Zhang, Yan-Yan Hu, Bin Zheng, Wei-Qiang Tan

**Affiliations:** 1 Department of Hand Surgery, The First Affiliated Hospital, Zhejiang University School of Medicine, Hangzhou, Zhejiang, China; 2 Department of Plastic Surgery, Sir Run Run Shaw Hospital, Zhejiang University School of Medicine, Hangzhou, Zhejiang, China; 3 Department of Plastic Surgery, The Fourth Affiliated Hospital, Zhejiang University School of Medicine, Yiwu, Zhejiang, China

**Keywords:** Wound healing, Truncated transforming growth factor-β type II receptor, Hepatocyte growth factor, Scar, Rat, Lentivirus

## Abstract

Wound scarring remains a major challenge for plastic surgeons. Transforming growth factor (TGF)-β plays a key role in the process of scar formation. Previous studies have demonstrated that truncated TGF-β type II receptor (t-TGF-βRII) is unable to continue signal transduction but is still capable of binding to TGF-β, thereby blocking the TGF-β signaling pathway. Hepatocyte growth factor (HGF) is a multifunctional growth factor that promotes tissue regeneration and wound healing. Theoretically, the combination of HGF and t-TGF-βRII would be expected to exert a synergistic effect on promoting wound healing and reducing collagen formation. In the present study, lentivirus-mediated transfection of the two genes (t-TGF-βRII/HGF) into fibroblasts *in vitro* and in a rat model *in vivo* was used. The results demonstrated that the expression of t-TGF-βRII and HGF in NIH-3T3 cells was successfully induced. The expression of both molecules significantly reduced collagen I and III expression, and also inhibited fibroblast proliferation. Furthermore, histological examination and scar quantification revealed less scarring in the experimental wound in a rat model. Moreover, on macroscopic inspection, the experimental wound exhibited less visible scarring compared with the control. Therefore, the present study demonstrated that the combination gene therapy of t-TGF-βRII and HGF promoted wound healing, with less scarring and more epithelial tissue formation, not only by suppressing the overgrowth of collagen due to its antifibrotic effect, but also by promoting tissue regeneration.

## Introduction

Scar formation during the process of wound healing remains a challenge for surgeons. Although medical technology has developed rapidly over the past decades, there are few reliable methods to inhibit scar formation. Conventionally, scarring has been considered a natural result of wound healing and is inevitable. As a result, excessive inhibition of scar formation may lead to delayed wound healing. Therefore, an optimal therapeutic approach must focus on suppressing scar formation as well as promoting wound healing.

Previous studies have demonstrated that transforming growth factor (TGF)-β plays a key role in the process of scar formation (1), not only by accelerating the proliferation and division of fibroblasts, but also by promoting collagen synthesis. Therefore, reduction of wound TGF-β levels or inhibition of the TGF-β signaling pathway may prevent scar formation (2,3). Truncated TGF-β type II receptor (t-TGF-βRII) is a TGF-βRII that has lost its serine- and glycine-rich fragments (4). t-TGF-βRII is thus unable to continue signal transduction, but is still capable of binding to TGF-β, thereby blocking the TGF-β signaling pathway (5). Hepatocyte growth factor (HGF) is a multifunctional growth factor that promotes mitosis, has anti-apoptotic, anti-fibrotic, and pro-angiogenic properties, whereas it inhibits fibrosis and generation of TGF-β in hepatic tissue and enhances collagenase activity (6). In theory, the combination of HGF and t-TGF-βRII would be expected to exert a synergistic effect on promoting wound healing and reducing collagen formation.

In the present study, both *in vitro* and *in vivo* models were used to test this hypothesis. The aim of the study was to determine whether the combination of HGF and t-TGF-βRII would be able to reduce the expression of collagen and cell proliferation *in vitro* and *in vivo*, hoping to provide a novel approach to gene therapy.

## Material and Methods

### Cell culture

NIH-3T3 mouse embryonic fibroblasts (Shanghai Institute of Biochemistry and Cell Biology, China) were cultured in Dulbecco's modified Eagle's medium (Gibco; Thermo Fisher Scientific, Inc., USA) supplemented with 10% fetal bovine serum. The cells were cultured for 4 days at 37°C in a humidified incubator with 5% CO_2_.

### Plasmid construction

The cDNA codes of human HGF and human TGF-βRII were obtained from PubMed (Bethesda, USA). The DNA was amplified and digested by Bam I and Not I enzyme, purified from agarose gel. A truncated human TGF-β type II receptor cDNA code was obtained by removing the sequence of signal transduction, as described previously (4). Plasmid vectors (pLV-EFla-EGFP, pLV-EFla-HGF-EGFP, and pLV-EFla-tTGF BRII-EGFP) were designed and synthesized by Invitrogen (Thermo Fisher Scientific Inc., China).

### Lentivirus package

293T cells (Thermo Fisher Scientific, Inc., USA) were seeded onto 10-cm plates at a density of 2.0×106/mL 24 h prior to transfection. Lentivirus was packaged according to the instructions of HIV package kits when the confluence reached 70–80%. Plasmid pMD2 and psPAX2 were packaged together with target plasmids with a ratio of 2:1:2. A homemade calcium phosphate transfection was used; briefly, the plasmids and calcium phosphate were mixed very well and incubated for 15 min, then added to HEK293T cells. The lentivirus was harvested 24 h post-transfection and the supernatant was filtered with 0.45 μM filters.

### NIH-3T3 cell transfection

NIH-3T3 cells were inoculated into 6-well plates at a density of 2.0×10^5^ cells per well. The groups were set as follows: empty virus group (empty lentivirus, 2 mL/well), t-TGF-βRII control group (t-TGF-βRII-lentivirus, 2 mL/well), HGF group (HGF-lentivirus, 2 mL/well), and experimental group (t-TGF-βRII-lentivirus, 1 mL/well and HGF-lentivirus, 1 mL/well). The calcium phosphate transfection method was used for transfection, and transfection efficiency and the morphology of NIH-3T3 was observed 24 and 48 h after transfection. The transfection efficiency was calculated from percentage of GFP+ cells and the average was 75% (65–85%).

### Cell viability test

The change in cell proliferation was observed with the Cell Counting Kit-8 (CCK-8) colorimeter (Dojindo Molecular Technologies, Inc., Japan). Briefly, 5000 cells were seeded into a 96-well plate and after 12-h incubation, 50 μL of TGF-β1 (800 pg/mL) was added to each well and culturing continued for other 48 h. CCK-8 assay followed the protocol.

### Western blot analysis

The protein expression of t-TGF-βRII, HGF, collagen I, and collagen III were assayed by western blotting. Total proteins were extracted from each group 96 h post-transfection. A total of 50 μg of protein from each sample was loaded onto a 7.5% SDS-PAGE gel and electrophoresed at 150 V for about 1 h. Proteins were then transferred to nitrocellulose membranes (0.45 μm, EMD Millipore, USA) that had been blocked with 5% bovine serum albumin in Tris-buffered saline for 1 h and washed with TBS-T buffer three times at room temperature. The blots were incubated with primary antibodies against human TGF-βRII (1:1,000, Cell Signaling Technology, Inc., USA), HGF (1:1,000, Abcam, USA), collagen type I, or collagen type III (1:1,000, Sigma-Aldrich; Merck KGaA, USA), or GAPDH (1:2,000, Cell Signaling Technology, Inc.) overnight at 4°C. The membranes were washed three times and then incubated with horseradish peroxidase-conjugated goat anti-rabbit IgG (1:5,000, Pierce; Thermo Fisher Scientific, Inc.) for 1 h at room temperature. All blots were developed using enhanced chemiluminescence reagents (Supersignal Dura Kit, Pierce; Thermo Fisher Scientific, Inc.) according to the manufacturer's instructions. The signals were captured on X-ray film.

### Animal models

Male Sprague-Dawley rats, 4–6 weeks old and weighing 225–275 g, were used in the present study (n=24). The following experimental procedure was done under anesthesia using 2% sodium pentobarbital at 35 mg/kg by intraperitoneal injection. A 3-cm (length) × 2-mm (width) rectangular deep incision was made on the dorsal skin, which included the panniculus muscle. A 3-cm (length) × 3-mm (width) × 5-mm (height) trimmed gelatin sponge was inserted into the wound. The rat thick linear scar model was established as described by Wu et al. (7). Forty-eight hours before the animal model was established, 250 μL of t-TGF-βRII-lentivirus and 250 μL of HGF-lentivirus were injected subcutaneously under one side of the pre-designed incisions (1×10^6^ pfu/mL, n=6). A total of 500 μL of t-TGF-βRII-lentivirus, HGF-lentivirus, empty lentivirus, or DMEM were injected subcutaneously on the other side (1×10^6^ pfu/mL, n=6). The samples were harvested 14 h post-transfection. The middle 5-mm portion of the scar was paraffin-embedded, sectioned, stained with hematoxylin and eosin, and observed under a microscope. All animal experiments were approved by the Laboratory Animal Center of Zhejiang University (SYXK 2013-0180).

### Histology and qualification of the scar area

After staining with hematoxylin and eosin, on day 14 tissue sections of the wounds were observed under a microscope (Nikon Corporation, Japan) at a fixed magnification (×100), and the images were recorded digitally onto a computer with the Image-Pro Plus 6.0 system (Media Cybernetics, USA). The relative area of the wound scar was defined by the ‘Irregular AOI' tool and then measured and recorded automatically by the ‘Count/Size' tool of Image-Pro Plus (Media Cybernetics, USA).

### Statistical analysis

Data were analyzed using the SPSS 13.0 software (SPSS Inc., USA). The data are reported as means±SD. Comparison among the groups was performed using analysis of variance followed by the least significant difference (LSD) and SNK (student-Newman-Keuls) tests. A P-value <0.05 was considered to indicate statistically significant differences.

## Results

### Higher expression of t-TGF-βRII and HGF was observed *in vitro* and *in vivo* following transfection

**Figure 1:**
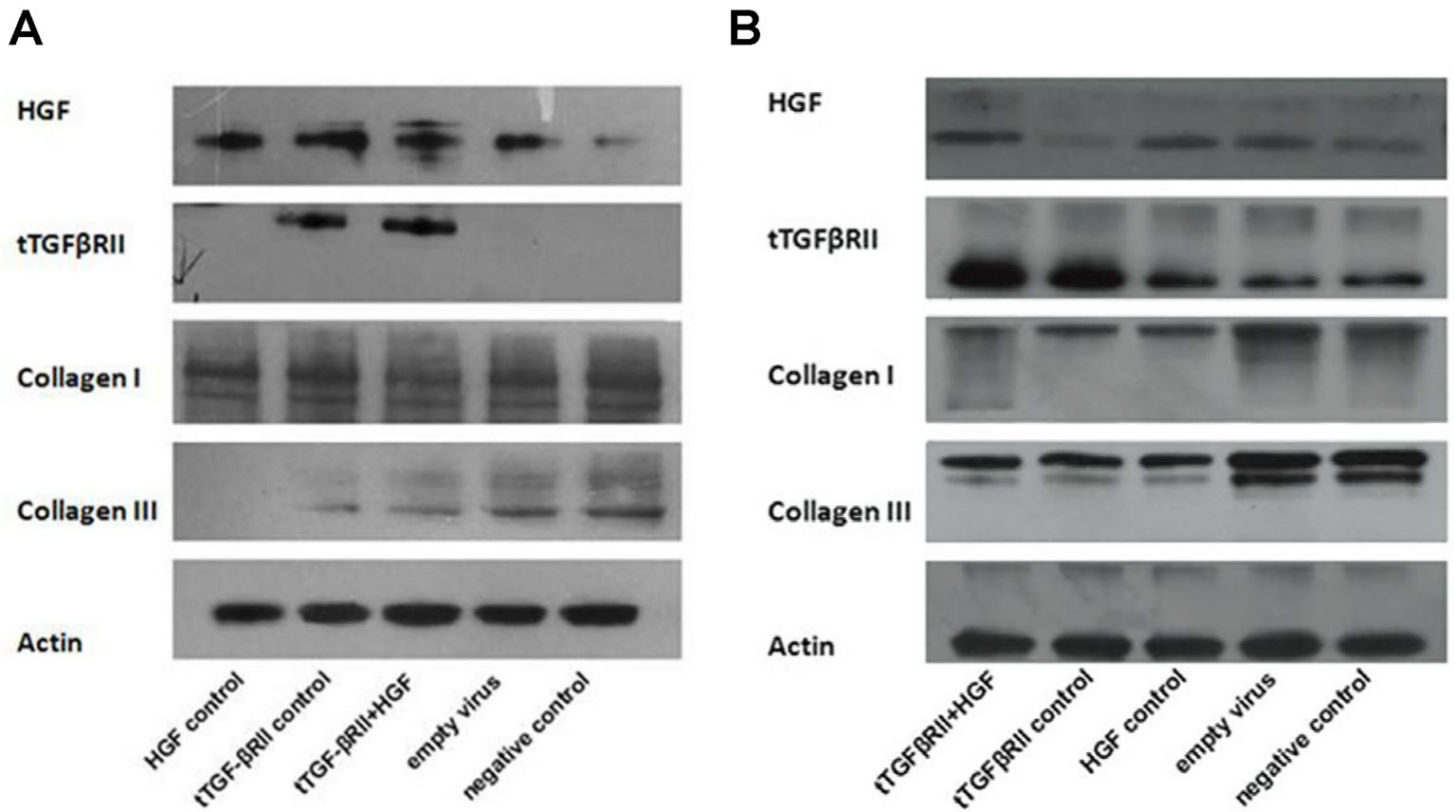
Expression of t-TGF-βRII, HGF, and collagen I and III *in vitro* and *in vivo*. **A**, In the *in vitro* experiment, the expression of collagen III was reduced in HGF control, t-TGF-βRII, and experimental groups compared with that in the blank and negative control groups. The result was most prominent in the HGF control group. **B**, In the *in vivo* experiment, the expression of collagen I and III was significantly reduced in the HGF control, t-TGF-βRII, and experimental groups after 14 days of treatment. t-TGF-βRII: truncated transforming growth factor-β type II receptor; HGF: hepatocyte growth factor.

The NIH-3T3 is a well-established cell line for the study of wound healing, and it was used to test our hypothesis in the present study. The expression of t-TGF-βRII in the experimental group and control group was found to be notably higher compared with that in other groups, *in vitro* as well as *in vivo* (Figure 1A and B). Moreover, the same result was observed for the expression of HGF, indicating that the transfection was successful, and the model could be used for further study.

### t-TGF-βRII reduced the expression of collagen I and III

Previous studies have demonstrated that TGF-β signaling plays a crucial role in the process of scar formation by accelerating the proliferation of fibroblasts and by promoting collagen synthesis; however, t-TGF-βRII inhibits these effects (1). We next tested whether t-TGF-βRII affects the expression of collagen. *In vitro* data results revealed that the expression of collagen I was markedly reduced in the experimental group. In addition, the expression of collagen III was reduced in the HGF control, t-TGF-βRII, and experimental groups, compared with that in the blank and negative control groups (Figure 1A and B).

### Transfection of t-TGF-βRII and HGF inhibited the proliferation of NIH-3T3 cells

**Figure 2:**
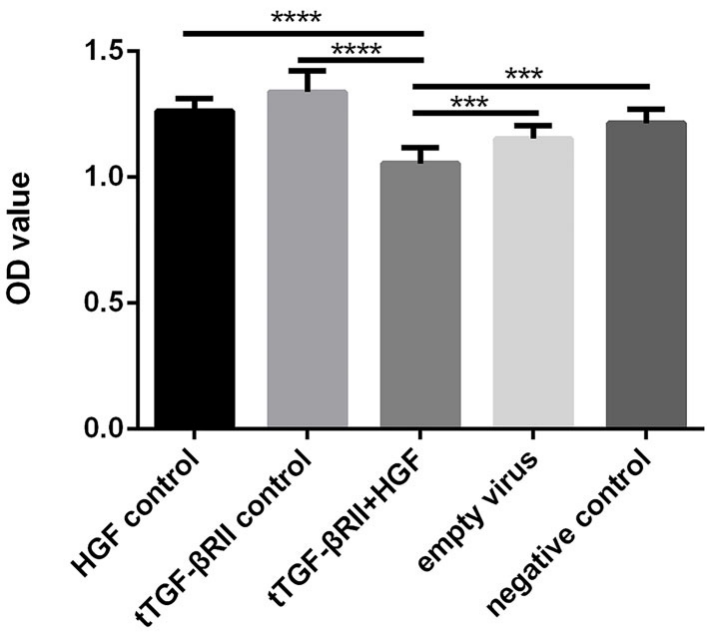
The proliferation of NIH-3T3 cells, which was quantified by Cell Counting Kit-8 assay, was significantly inhibited by the transfection of t-TGF-βRII/HGF-lentivirus, compared with that in the control groups (P<0.05). Data are reported as means±SD. ***P<0.001 and ****P<0.0001 (least significant difference (LSD) test). t-TGF-βRII, truncated transforming growth factor-β type II receptor; HGF, hepatocyte growth factor.

The proliferation of cells transfected with t-TGF-βRII/HGF lentivirus was significantly inhibited compared with the control group and single transfected control (Figure 2).

### Transfection of t-TGF-βRII and HGF inhibited wound scarring and promoted tissue repair

**Figure 3:**
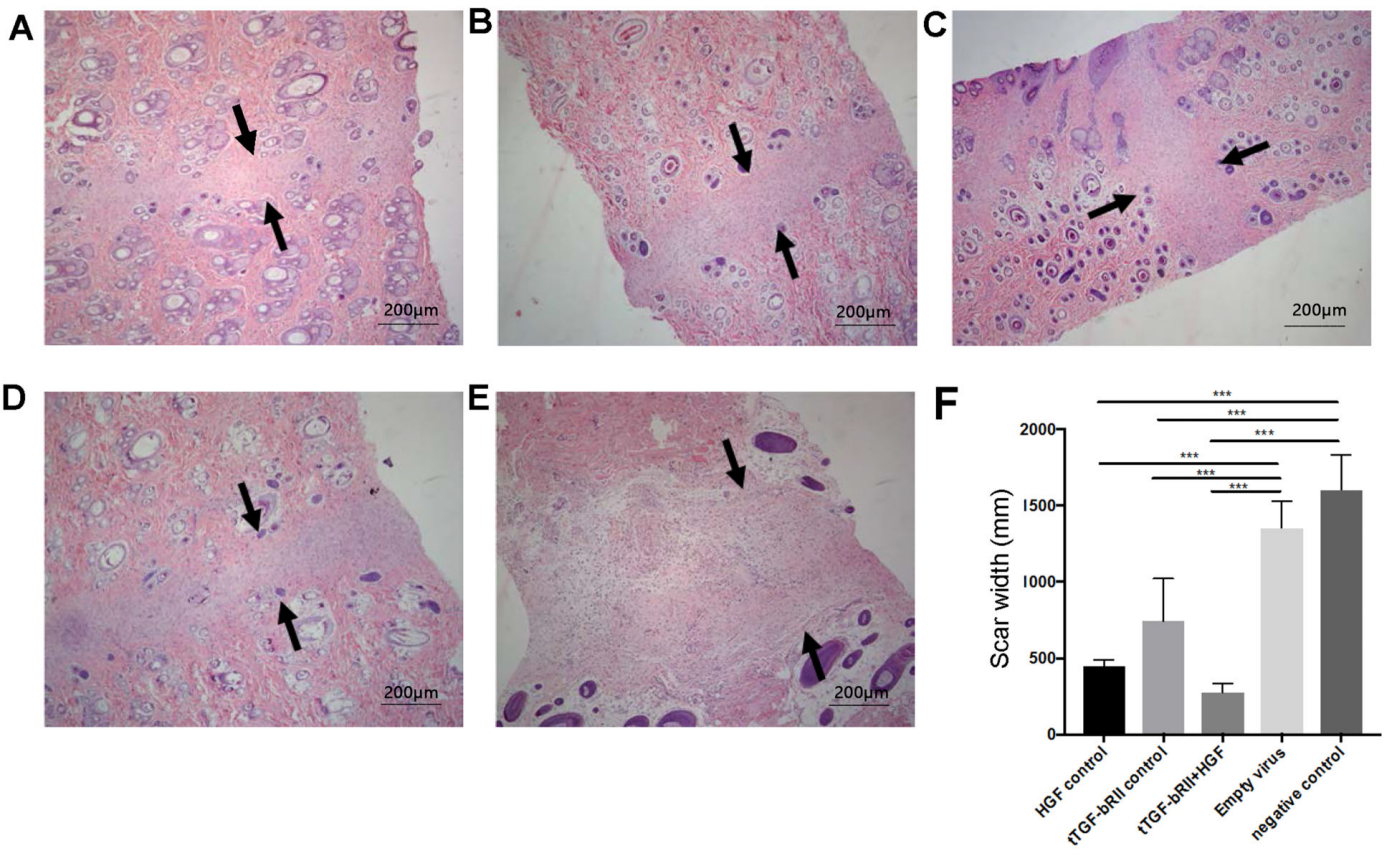
Scar width in each group. Histological examination revealed that the width of the scar was narrower in the experimental wound (hematoxylin and eosin staining; original magnification: ×100, bars: 200 μm). Arrows indicate the scar width. **A**, Experimental group (t-TGF-βRII+HGF); **B**: t-TGF-βRII group; **C**: HGF group; **D**: empty virus group; **E**: blank group. **F**, Summary of scar width. Data are reported as means±SD. ***P<0.001 (Student-Newman-Keuls test). t-TGF-βRII: truncated transforming growth factor-β type II receptor; HGF: hepatocyte growth factor.

**Figure 4:**
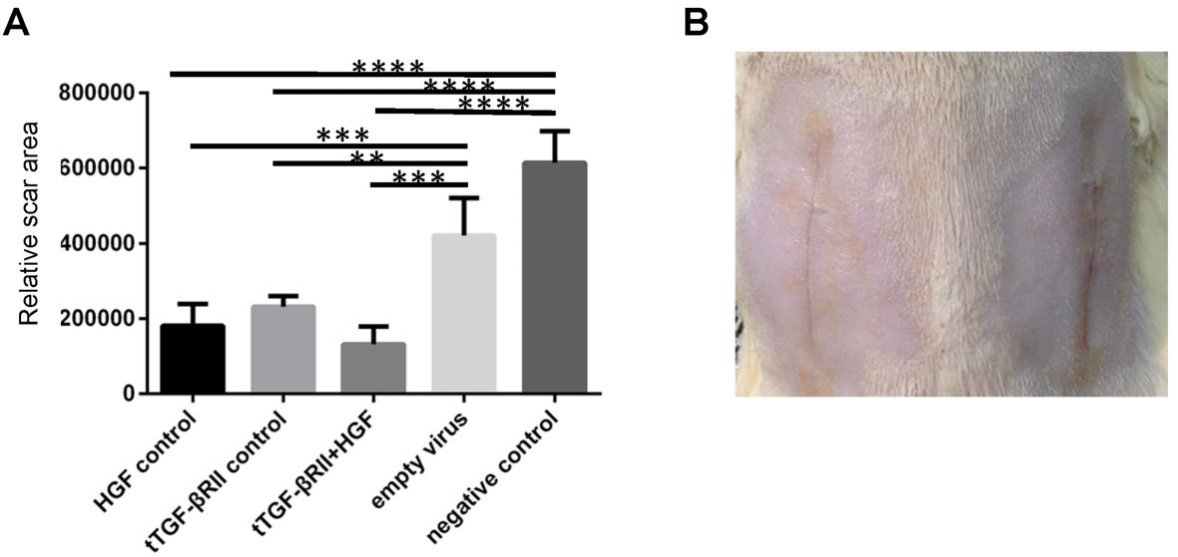
Relative scar area in each group. **A**, Quantification of the relative wound scar area revealed that the wounds in the experimental (t-TGF-βRII+HGF), HGF control, and t-TGF-βRII groups healed with less scarring compared with those in the empty virus and blank groups. The result was most prominent in the experimental group. Data are reported as means±SD. **P<0.01, ***P<0.001, ****P<0.0001 (Student-Newman-Keuls test). **B**, On macroscopic inspection, the treated wound (left side) exhibited less visible scarring compared with the control wound (right side) at 14 days after wounding. t-TGF-βRII: truncated transforming growth factor-β type II receptor; HGF: hepatocyte growth factor.

Our *in vitro* results indicated that the transfection of t-TGF-βRII and HGF inhibited cell proliferation. To further investigate these effects, they were next tested in an established rat model. Both molecules were highly expressed in the rat model (Figure 1B). Histological examination revealed that the width of the scar was narrower compared with that in the untreated group (Figure 3A-F). Quantification of the wound scar area demonstrated that the wounds in the experimental (t-TGF-βRII + HGF), HGF control, and t-TGF-βRII groups healed with less scarring compared with those in the empty virus and blank groups. The result was most prominent in the experimental group (Figure 4A). On macroscopic inspection, the treated wounds exhibited less visible scarring compared with the control wounds at 14 days after wounding (Figure 4B).

The above-mentioned results indicated that the combination of t-TGF-βRII and HGF mediated by lentivirus can be successfully transfected into fibroblasts. Fibroblast proliferation and fibrosis can be inhibited by the cotransfection of HGF and t-TGF-βRII. Therefore, combination gene therapy with t-TGF-βRII and HGF promoted wound healing with less scarring and more epithelial tissue formation, not only by suppressing the overgrowth of collagen, due to its antifibrotic effect, but also by promoting tissue regeneration.

## Discussion

Wound healing is a complicated process that has only started to become elucidated (8). Scar formation has been conventionally considered a natural event during the process of wound healing. A number of studies have reported that TGF-β signaling plays a fundamental role in pathological fibroproliferation and abnormal scar formation (1,9,10). During the process of wound healing, TGF-β binds to and activates specific receptors located on the cell membrane. The ligand TGF-β assembles a receptor complex that activates the Smad proteins, and these proteins assemble multi subunit complexes that regulate transcription (3).

Strategies for scar reduction have been adopted based on targeting of the TGF-β signaling pathway. Shah et al. (11) demonstrated that wounds in adult rodents could be modified to reduce scar formation using a TGF-β neutralizing antibody, providing direct evidence that manipulating wound TGF-β can inhibit scarring. In addition, wounds in TGF-β knockout mice heal with less formation of granulation tissue and faster epithelialization compared with control mice (12). Numerous studies have demonstrated that blocking the TGF-β signaling pathway may be more effective in inhibiting TGF-β-mediated scar formation (2–4,13,14).

Systemic pharmacological inhibitors of collagen synthesis have been used clinically, with significant systemic adverse effects (15). Therefore, it is necessary to develop a method for inhibiting scar formation without inhibition of wound healing. HGF is a multifunctional growth factor, which can promote mitosis, has anti-apoptotic, anti-fibrotic, and pro-angiogenic properties, inhibits fibrosis and generation of TGF-β in hepatic tissue, and enhances collagenase activity (6). It has been suggested that HGF prevents fibrosis in liver and lung injury models (16-18), whereas other studies revealed that HGF prevents fibrosis after injury or surgery and facilitates tissue regeneration in wounds (6,19,20).

The advantages of gene therapy are the higher efficiency, fewer side effects, and a long expression time in wounds. Ozawa et al. (21) used combination gene therapy with HGF and t-TGF-βRII for rat liver cirrhosis following partial hepatectomy and suggested that gene therapy may increase the possibility of hepatectomy in a cirrhotic liver by improving fibrosis, hepatic function, and hepatocyte regeneration. In the present study, a similar strategy was applied for gene therapy of wound scarring. Incisional wounding was performed in rats and double gene (t-TGF-βRII/HGF) complexes were successfully transfected into NIH-3T3 mouse embryonic fibroblasts by lentivirus to study the effectiveness of blocking TGF-β signaling combined with HGF.

In our *in vitro* experiments, the expression of collagen I and III in the experimental group was found to be significantly reduced compared with that in the blank and negative control groups. The proliferation of NIH-3T3 cells, which was quantified by the CCK-8 assay, was significantly inhibited by the transfection of t-TGF-βRII/HGF-lentivirus. *In vivo* gene therapy in the rat model demonstrated that the expression of collagen I and III in the experimental group was significantly reduced compared with that in the control groups.

Based on the above-mentioned data, it may be concluded that fibrosis and wound scarring were inhibited by the cotransfection of HGF and t-TGF-βRII. Furthermore, histological examination and qualification revealed that the width of the scar was markedly narrower in the experimental group compared with that in the control groups, including the single t-TGF-βRII control group. These findings support that the combination gene therapy of t-TGF-βRII and HGF promoted wound healing with less scarring and more epithelial tissue formation, not only by suppressing the overgrowth of collagen due to its antifibrotic effect, but also by promoting tissue regeneration. However, HGF expression needs to be further optimized and, in addition, since we used lentiviral vector to deliver two target genes, the effects of continued expression of these two molecules need to be further investigated. A vector with on/off switch features can be also tested in this animal model.
